# Unveiling the Effects of the COVID-19 Pandemic on Lung Cancer Surgery

**DOI:** 10.3390/medicina60060964

**Published:** 2024-06-11

**Authors:** Gabriel Veniamin Cozma, Calin Muntean, Alaviana Monique Faur, Vasile Gaborean, Ioan Adrian Petrache, Catalin Vladut Ionut Feier

**Affiliations:** 1 Thoracic Surgery Research Center, “Victor Babeş” University of Medicine and Pharmacy Timişoara, Eftimie Murgu Square No. 2, 300041 Timişoara, Romania; gabriel.cozma@umft.ro (G.V.C.); vasile.gaborean@umft.ro (V.G.); ioan.petrache@umft.ro (I.A.P.); 2 Department of Surgical Semiology, Faculty of Medicine, “Victor Babeş” University of Medicine and Pharmacy Timişoara, Eftimie Murgu Square No. 2, 300041 Timişoara, Romania; 3 Medical Informatics and Biostatistics, Department III-Functional Sciences, “Victor Babeş” University of Medicine and Pharmacy Timişoara, Eftimie Murgu Square No. 2, 300041 Timişoara, Romania; 4 Faculty of Medicine, “Victor Babeş” University of Medicine and Pharmacy Timişoara, 300041 Timişoara, Romania; alaviana.faur@student.umft.ro; 5 First Discipline of Surgery, Department X-Surgery, “Victor Babeş” University of Medicine and Pharmacy Timişoara, Eftimie Murgu Square No. 2, 300041 Timişoara, Romania; catalin.feier@umft.ro; 6 First Surgery Clinic, “Pius Brinzeu” Clinical Emergency Hospital, 300723 Timişoara, Romania

**Keywords:** COVID-19 pandemic, lung cancer surgery, length of hospitalization, surgical postponement

## Abstract

The aim of this study is to investigate the impact of the COVID-19 pandemic on the surgical treatment of lung cancer patients. Data from patients who underwent surgery during the pandemic were analyzed and compared to pre-pandemic and post-pandemic periods. Multiple parameters were examined, and their changes yielded significant results compared to other periods of the study. The statistical analysis revealed a significant decrease in the number of surgical interventions during the pandemic (*p* < 0.001), followed by a significant rebound thereafter. During this period, there was a significant increase in the T stage of cancer compared to both pre-pandemic and post-pandemic periods (*p* = 0.027). Additionally, the mean Charlson comorbidity index score was significantly higher during the pandemic compared to the pre-pandemic period (*p* = 0.042). In this crisis period, a significant decrease was recorded in both the total hospitalization duration (*p* = 0.015) and the pre-operative hospitalization duration (*p* = 0.006). These findings provide evidence of significant changes in clinical and therapeutic strategies applied to lung cancer surgery patients during the study period. The pandemic has had a substantial and complex impact, the full extent of which remains to be fully understood.

## 1. Introduction

Lung cancer stands as the most prevalent and fatal malignancy globally across genders, as reported by Globocan 2022 [1]. In Romania, lung cancer ranks third among neoplastic pathologies, emerging as the second most common cancer in males and the fourth in females, with a mortality rate of 26.6 per 100,000 individuals, regardless of gender, as per the Global Cancer Observatory (https://gco.iarc.fr/en (accessed on 22 April 2024)) [2].

The unprecedented COVID-19 pandemic has necessitated governmental directives that have significantly altered screening and therapeutic protocols concerning oncological malignancies worldwide. These directives, globally, prioritized COVID-19 cases, postponing surgical procedures deemed low risk, requiring SARS-CoV-2 testing before hospital admissions, and minimizing hospitalization durations to alleviate the strain on the medical system and intensive care units [3]. Regarding lung cancer, the therapeutic approach underwent meticulous review due to the virus’s primary localization in the same organ prone to neoplastic degeneration [4]. Some studies have highlighted a notable increase in estimated avoidable cancer-related deaths due to diagnostic and treatment delays attributable to the COVID-19 pandemic [5,6].

During the pandemic, a judicious assessment of the risk–benefit ratio for lung cancer treatment was paramount. However, there is a paucity of studies adequately illustrating surgical practices for this condition in Romania [7]. This research endeavors to address this gap by conducting a comprehensive analysis and interpretation of the clinical and pathological aspects of lung cancer patients amidst the COVID-19 pandemic. Focusing on this distinctive temporal context, this study aims to meticulously monitor and evaluate the dynamic changes observed in various parameters compared to both the pre-pandemic and post-pandemic periods in patients who underwent surgery for lung cancer treatment at the Thoracic Surgery Clinic of the Emergency Municipal Hospital of Timisoara.

## 2. Materials and Methods

This study aims to evaluate patients who underwent pulmonary lobectomy for lung cancer treatment at the Thoracic Surgery Clinic of the Municipal Clinical Hospital in Timisoara. Thus, the data of 106 patients undergoing surgical intervention between 26 February 2018 and 25 February 2024 were analyzed.

To highlight the impact that the COVID-19 pandemic had on the treatment and management of these patients, the study period was divided into three groups:

- 26 February 2018 to 25 February 2020, representing the pre-pandemic group.
- 26 February 2020 to 25 February 2022, representing the pandemic group.
- 26 February 2022 to 25 February 2024, representing the post-pandemic group.

The date of February 26 was not randomly chosen. On 26 February 2020, the first positive COVID-19 case was confirmed in Romania, and on 8 March 2022, all restrictions imposed by authorities were lifted. Thus, in order to gain an overview of the impact of the pandemic on the surgical treatment of these patients, three equally long periods were selected.

For this study, multiple inclusion criteria were established. Specifically, only patients who underwent lobectomy of one of the lung lobes during the six-year study period were included. Patients with metastases from other types of cancer or patients in stage IV of non-small-cell lung cancer were excluded. Furthermore, only patients for whom the histopathological result of the excised tumor indicated a type of non-small-cell lung cancer, namely, adenocarcinoma or squamous cell carcinoma, were considered. During the analysis and design of our database, it was identified that large-cell carcinoma accounted for fewer than 4% of cases. This low percentage was the reason why the presence of such a histopathological diagnosis was considered an exclusion criterion.

Considering that this study included the period of the pandemic, additional inclusion criteria were devised to ensure a comprehensive evaluation. It is well known that active infection with the SARS-CoV-2 virus significantly impacts the prognosis and post-operative course of patients with lung cancer [8,9,10]. Therefore, since this study focuses on the pandemic’s impact on the management of these patients, rather than the direct consequences of SARS-CoV-2 infection, patients who had a prior infection with this virus before surgery or during hospitalization were excluded from this study. Moreover, patients who exhibited typical COVID-19 symptoms upon admission or within 7 days prior to admission were not eligible. Finally, only patients who underwent an RT-PCR test for SARS-CoV-2 detection upon admission and obtained a negative result, or those who underwent a test with negative results within 24 h before admission, were considered. Ethical approval for data collection was obtained from the Hospital Commission (No. E-2633/19 April 2024).

Once the inclusion criteria were met, multiple parameters of these patients were investigated. Data such as gender, age, environment of origin, and smoking status were considered. Given that only patients who underwent lobectomy were analyzed, tumors that did not extend beyond one lung lobe were taken into account. The results section also presents the localization of these tumors based on lung lobes (upper, middle, or lower lobes of the right lung and upper or lower lobes of the left lung). Post-operative complications, such as air leak occurrence, time spent in the ICU (intensive care units), and duration of surgical intervention, were monitored. The patients’ comorbidities were assessed using the Charlson comorbidity index. Regarding histopathological type, this study predominantly focused on patients with non-small-cell lung cancer. Therefore, the frequency of adenocarcinoma or squamous cell carcinoma was also analyzed, with the number of patients with large-cell carcinoma being extremely limited and thus not included in the analysis. Tumor size was another parameter analyzed, along with the stage of tumor invasion (T), lymph node involvement (N), presence or absence of metastasis (M), and consequently, the stage of the disease.

In addition, due to the unique circumstances generated by this pandemic and the risk of SARS-CoV-2 infection associated with prolonged exposure to the medical environment, we also analyzed the total duration of hospitalization, along with pre-operative and post-operative hospital stay durations and the duration of surgery.

For statistical analysis and obtaining results, we utilized IBM SPSS Statistics 25 software for Windows (IBM, Armonk, NY, USA). Descriptive statistics were employed for numerical variables, encompassing the calculation of measures of central tendency and dispersion. Regarding categorical variables, frequency tables and percentages were utilized to illustrate variations across the study periods.

To facilitate comparisons between two independent samples, the Mann–Whitney test was employed. For comparisons involving more than two samples, we utilized the ANOVA test, allowing for simultaneous analysis of variance among multiple groups. Additionally, the Chi-square test was employed to assess the association between categorical variables and to highlight differences in proportions. The Pearson correlation coefficient (r) was used to identify correlations among the study variables.

In our analyses, statistical significance was defined as *p* < 0.05 for all applied statistical tests, indicating results unlikely to have occurred by random chance.

## 3. Results

For this study, we analyzed data from patients who underwent elective surgical intervention for the treatment of lung cancer. We considered the data of patients operated on during the pandemic period between 26 February 2020 and 25 February 2022. The obtained results were compared with the pre-COVID and the post-COVID period.

### 3.1. Patient Demographics

Data from 106 patients who met the inclusion criteria and underwent surgery at the Thoracic Surgery Clinic of the Municipal Hospital in Timisoara were analyzed. During the pandemic period, 19 surgeries (17.9%) were performed; while in the pre-pandemic period, there were 46 surgeries (43.4%) and in the post-pandemic period, 41 surgeries (38.7%) were conducted. Upon applying the Chi-square test to highlight the differences in proportions among the three periods, a *p*-value of <0.001 was obtained.

The average age, gender distribution, and patients’ environment of origin are presented in Table 1.

In terms of the proportion of male patients during the pandemic period, significant differences were observed compared to the post-pandemic period, with a *p*-value of 0.035 (36.8% vs. 65.9%).

This study revealed an association between smoking status and gender (*p* = 0.02), with 50.8% of male patients undergoing surgery being smokers compared to only 20.9% of female patients. Furthermore, an association between age and smoking status was also identified (*p* = 0.048), with smokers having an average age of 59.98 ± 7.41 compared to non-smokers with an average age of 63.34 ± 8.96.

### 3.2. Patients and Tumor Characteristics

Table 2 depicts the tumor location, size, and histopathological type of the analyzed tumors across the three time periods.

Smoking patients exhibited similar percentages for both adenocarcinoma (48.8%) and squamous cell carcinoma (5.2%). However, an association was identified between non-smoking status and histopathological type (*p* = 0.041), with non-smokers predominantly presenting adenocarcinoma (68.8%), compared to only 31.3% with squamous cell carcinoma.

Furthermore, histopathological-type adenocarcinoma was reported in 76.2% of male patients compared to only 50.8% in female patients, indicating an association between gender and histopathological type (*p* = 0.009).

The variations of TNM and stage of cancer are presented in Table 3.

For assessing the comorbidities of patients in the study, the Charlson comorbidity index was utilized. During the pandemic period, the average value was 4.47 ± 1.21, while in the pre-pandemic period, it averaged 5.22 ± 1.47. In the post-pandemic period, the average was 4.85 ± 0.98. Statistical tests to identify significant differences among the three periods resulted in a *p*-value of 0.086. Analyzing differences between the pre-pandemic and pandemic periods regarding the Charlson index mean, the statistical results generated a *p*-value of 0.042.

An association between patients’ gender and the Charlson comorbidity index was observed (*p* = 0.014). Male patients had an average index value of 5.19 ± 1.28, while female patients had an average of 4.58 ± 1.20.

Another parameter analyzed was the duration of ICU hospitalization. During the pandemic, patients spent an average of 2.47 ± 1.3 days in the ICU compared to 2.54 ± 1.78 days in the initial period of the study and 2.34 ± 1.17 days in the post-pandemic period. Statistical tests to identify differences among the three periods resulted in a *p*-value of 0.81.

### 3.3. Hospitaly Stay and Post-Operative Complciations

Statistical analysis revealed significant differences between the pandemic and post-pandemic periods regarding the mean duration of the three hospitalization periods. A *p*-value of 0.001 was found for the total hospitalization duration between the two periods, *p* < 0.001 for pre-operative hospitalization duration, and *p* = 0.033 for total post-operative hospitalization duration. In the pre-pandemic period, a significant difference (*p* < 0.001) was observed in pre-operative hospitalization duration.

Variations of these parameters are presented in Table 4.

Furthermore, direct correlations were found between the total hospitalization duration and the following:

- Surgical intervention duration (*p* = 0.025; r = 0.3);
- Number of days spent in intensive care (*p* < 0.001; r = 0.440).

Similarly, direct correlations were observed between post-operative hospitalization duration and the following:

- Surgical intervention duration (*p* = 0.019; r = 0.314);
- Number of days spent in intensive care (*p* < 0.001; r = 0.498).

Regarding patients with air leaks, a significant proportion hailed from rural areas (52.4% vs. 31.3%, *p* = 0.03) and presented with more advanced tumor stage (*p* = 0.018) and disease stage (*p* = 0.022). This complication was reported in 8 cases (42.1%) during the pandemic period, 15 cases (32.6%) in the pre-pandemic period, and 19 cases (46.3%) in the post-pandemic period. The chi-square test yielded a *p*-value of 0.413 for differences in proportions among the three periods.

## 4. Discussion

The COVID-19 pandemic was a global health crisis that caused major disruptions to society and healthcare systems worldwide, resulting in significant changes in medical practice and surgical treatment of cancers [11,12]. In this study, we aimed to evaluate the impact of the pandemic on patients undergoing surgery for lung cancer by comparing the pandemic period with the pre- and post-pandemic periods. Our analysis involved examining the data of 106 patients operated at the Thoracic Surgery Clinic of the Municipal Hospital in Timișoara to better understand the trends and changes that occurred during this tumultuous period.

Various oncological and surgical organizations (European Society for Medical Oncology ESMO, Thoracic Surgery Outcomes Research Network, British Thoracic Society Lung Cancer) have developed sets of recommendations and guidelines for the assessment of patients with lung cancer scheduled for surgical interventions and for their management during the crisis period [13,14,15].

This study reveals a significant decrease in the number of elective lobectomies performed for the treatment of lung cancer during the pandemic period, with a decrease from 46 cases to 19, representing a decrease of 58.6% compared to the previous period. These declines primarily occurred due to the postponement of all elective oncological surgical interventions in the early stages of the pandemic, as well as due to government-imposed restrictions [16]. The decrease in the number of surgeries has been reported globally, with a hospital in the US also reporting a decrease of 50.4% compared to the previous period [17]. Significant differences have been evidenced in Queensland [18], as well as in countries such as Japan [19], Sweden [20], or England [21], where a significant decline in the number of surgical interventions performed has also been reported.

It is well known that delays in the treatment of cancerous pathologies have a negative impact on patient prognosis [22,23]. During this period, global lung cancer screening has significantly decreased, with countries such as Denmark [24], the Netherlands [25], England [20], and other Nordic countries reporting decreases of up to 20% during the pandemic [21]. The decrease in screening inevitably leads to a reduction in the number of interventions, and during this disrupted period, it was significantly influenced by both government-imposed restrictions and patients’ fear of visiting hospitals. A study conducted on lung cancer patients participating in clinical trials during the SARS outbreak revealed that 64% of them avoided visiting a hospital out of fear of infection, and 4% chose to discontinue treatment due to infection-related concerns. These figures highlight the psychological and emotional impact of the pandemic on cancer patients, who face a fragile balance between the need for medical care and the fear of being exposed to the virus [26].

The uniqueness of this study lies in its presentation of the post-pandemic situation. It demonstrates an increase in the number of interventions during the post-pandemic period from 19 cases to 41 cases, an increase of over 215%, indicating a return to the pre-pandemic situation, at least in this regard. Our findings are consistent with similar studies conducted in other countries, which also highlighted a return to normalcy regarding the number of oncological surgical interventions after the peak of the pandemic. This observation is encouraging and suggests an effective adaptation of the medical system to the conditions imposed by the pandemic. With the lifting of restrictions and a significant reduction in the number of positive COVID-19 cases, screening programs and surgical interventions have resumed their normal course, with a study from Australia showing an increase of over 170% in the number of surgical interventions for lung cancer following the lifting of restrictions [18].

Regarding age and place of origin, no significant differences were reported among the three periods. These results are consistent with the literature, where in most studies, these variables did not significantly change throughout the pandemic [17,19,24,27,28,29,30]. However, Kirk et al. [18] identified that patients undergoing surgery during the pandemic exhibited significantly higher ages (*p* = 0.006) compared to the pre-pandemic period.

An intriguing finding was the reduction in the proportion of male patients during the pandemic compared to the pre- and post-pandemic periods, along with an association of the male gender with a higher Charlson index. Male patients were more reluctant to seek hospital care during the pandemic, resorting to medical services only in the case of more aggressive symptoms, exacerbated by the presence of multiple comorbidities. Paradoxically, across the entire study, the Charlson index did not differ significantly among the three periods, akin to findings from a study conducted in New York at the onset of the pandemic, which showed no significant differences between pre-pandemic and pandemic groups regarding individual comorbidities, except for a higher incidence of hypertension in the pandemic group (*p* = 0.008) [27].

It is well-established that advanced T, N, and M stages represent negative prognostic factors for patients with non-small-cell lung cancer [31,32]. Our study highlights significant differences in terms of T stage and its variation across the three periods. T2–T4 stages comprised 89.7% of cases during the pandemic compared to only 65.2% during the pre-pandemic period. However, it is notable that as the pandemic waned, things gradually returned to normal, with early stages enjoying a significant increase in proportions, accounting for 48.9% of T2–T4 stages. This discrepancy between the pandemic and post-pandemic periods has been observed worldwide [17,18]. Kato et al. [19] reported a significant shift in the distribution of stage I diseases during the pandemic, with a notable decrease in stage IA1 cases and a substantial increase in stage IA3 and IB incidences compared to previous years. Their study revealed that within stage I tumors, both tumor and invasive sizes were significantly larger in the pandemic cohort than in the pre-pandemic cohort (tumor size: *p* = 0.031; invasive size: *p* < 0.001). An average increase in tumor size was also reported in our study (4.01 ± 1.41 vs. 3.58 ± 1.84), attributed to both delayed interventions and governmental restrictions as well as patients’ fear of engaging with the healthcare system, presenting only in cases of more severe symptoms, inherently linked to advanced stage and increased tumor size.

One of the most common post-operative complications in the surgical treatment of patients with non-small-cell lung cancer is represented by air leaks [33,34,35]. Although no significant differences were reported in this study among the three periods, associations were detected between the presence of these complications and both the more advanced T stage (*p* = 0.018) and the more advanced cancer stage (*p* = 0.022). We mentioned earlier that the pandemic presented a significant increase in T stages, which represent a negative prognostic factor; from this, it is clear that the pandemic period represented a significant risk factor for the prognosis of patients and early post-operative evolution. Literature studies support this idea, with Fraser et al. showing a significant increase in post-operative complications during the pandemic (45.7% vs. 36.8%), as well as a significantly higher proportion of patients admitted to intensive care units post-operatively [28].

Last but not least, the duration of hospitalization was investigated and analyzed. Surgeons aimed to shorten the time patients spent in the hospital, especially to reduce the risk of contamination with the novel coronavirus and minimize contact with medical staff, who presented an additional risk of exposure to the virus. This study shows a significant decrease both in the total duration of hospitalization (*p* = 0.015) and in the pre-operative hospitalization duration (0.006). In a study from Japan [19], the mean hospitalization duration was 6 days, without a significant change compared to the pre-pandemic period; but in Australia, a significant decrease in both pre-operative and post-operative hospitalization duration was reported [18]. Hospitals in Europe significantly reduced the duration of hospitalization during the pandemic [21,25], and those in the US followed the same trend [36]. In our study, it should be noted that with the resumption of normal activity, both pre-operative and post-operative hospitalization periods increased. During the pandemic, 1548 patients were admitted to this clinic, compared to 2028 patients in the previous period, and in the post-pandemic period, this number reached 2153. Thus, the increased number of patients automatically led to a prolongation of both pre-operative and post-operative waiting times. Furthermore, in the last period of the study, a significant increase in the average duration of surgical intervention was observed, a duration which correlated with the number of days patients spent in the hospital (*p* = 0.025; r = 0.3). This is due to more complex surgical interventions requiring longer operating times, as well as prolonged patient recovery periods.

Therefore, this pandemic has had significant effects on the clinical and therapeutic management of patients with lung cancer. International studies show that delaying treatment is not a solution; a meta-analysis demonstrates that survival decreases when surgical intervention is delayed by 42 days from staging according to The National Institute for Health and Care Excellence (available at https://www.nice.org.uk (accessed on 20 April 2024)) [37]. Additionally, Khorana and colleagues [38] have shown an increase in cancer-related deaths for each week of delay in initiating cancer treatment. Furthermore, these investigators found that delays in therapy of 6 weeks or more resulted in an absolute increase of 13% in lung cancer mortality at 5 years.

### Study Limitations

Our study, despite its merits, must be analyzed in the context of potential limitations. It was a retrospective study conducted at a single medical institution, and the relatively small sample size could affect the generalizability of the results. Additionally, being a study based on a state-level database, it is limited to the records of patients who underwent surgical interventions; thus, it is unable to capture the entire population of patients with lung cancer. Furthermore, the influence of changes in social paradigms on healthcare-seeking behavior during the pandemic was not directly quantified. With the imposed restrictions and encouragement of isolation, a significant number of patients may have postponed or even avoided seeking necessary medical treatment. Factors such as fear of contracting COVID-19, low confidence in the healthcare system, misinformation, and encouragement of isolation in cases where respiratory symptoms were detected could significantly influence the diagnosis and treatment of lung cancer. Despite these limitations, the uniqueness of this study lies in the inclusion of a post-pandemic period, with significant results that could serve as an important starting point for extensive research into creating protocols for managing patients with this condition in emergency situations, without significantly affecting their medium- and long-term prognosis.

## 5. Conclusions

The COVID-19 pandemic has had a significant impact on surgical practice in the treatment of lung cancer. During this period, we witnessed a substantial decrease in the number of surgical interventions, and patients presented with more advanced stages. These changes are the result of factors such as government-imposed restrictions, reduced access to screening, and patient anxiety about the risk of infection. However, the gradual resumption of surgical activity and the implementation of safety measures have led to an increase in the number of treated patients. It is essential to monitor the evolution of these changes carefully and to evaluate their long-term impact on the management of lung cancer.

## Figures and Tables

**Table 1:** Patients demographics.

Variables	Pre-Pandemic	Pandemic	Post-Pandemic	*p*
Gender Male Female	29 (63%) 17 (37%)	7 (36.8%) 12 (63.2%)	27 (65.9%) 14 (34.1%)	0.083
Age (years, M ± SD)	60.76 ± 8.13	61.63 ± 8.77	63.66 ± 7.85	0.251
Environment Urban Rural	27 (58.7%) 19 (41.3%)	14 (73.7%) 5 (26.3%)	23 (56.1%) 18 (43.9%)	0.412
Smokers Yes No	20 (43.5%) 26 (56.5%)	6 (31.6%) 13 (68.4%)	15 (36.6%) 26 (63.41%)	0.629

**Table 2:** The variation of tumor location, type, and size.

Variables	Pre-Pandemic	Pandemic	Post-Pandemic	*p*
Tumor location Right Lung Upper lobe Middle lobe Lower lobe Left Lung Upper lobe Lower lobe	5 (10.9%) 5 (10.9%) 18 (39.1%) 10 (10.9%) 8 (17.4%)	4 (21.1%) 1 (5.3%) 4 (21.1%) 5 (26.3%) 5 (26.35)	10 (24.4%) 2 (4.9%) 7 (17.1%) 15 (36.6%) 7 (17.1%)	0.257
Tumor type Adenocarcinoma Squamous cell Carcinoma	22 (47.8%) 24 (52.2%)	13 (72.2%) 5 (27.8%)	29 (70.7%) 12 (29.3%)	0.05
Tumor size (cm, M ± SD)	3.58 ± 1.84	4.01 ± 1.41	3.21 ± 1.52	0.26

**Table 3:** Variations of TNM and stage across the three periods of time.

Variables	Pre-Pandemic	Pandemic	Post-Pandemic	*p*
T1a T1b T1c T2a T2b T3 T4	3 (6.5%) 9 (19.6%) 4 (8.7%) 13 (28.3%) 10 (21.7%) 2 (4.3%) 3 (6.5%)	0 (0%) 1 (5.3%) 1 (5.3%) 7 (36.8%) 3 (15.8%) 5 (26.3%) 1 (5.3%)	1 (2.4%) 8 (19.5%) 12 (29.2%) 8 (19.5%) 2 (4.9%) 4 (9.8%) 4 (9.8%)	0.027
N0 N1 N2	33 (75%) 9 (20.5%) 2 (4.5%)	13 (76.5%) 2 (11.8%) 2 (11.8%)	30 (73.1%) 6 (14.6%) 3 (7.3%)	0.762
Stage I A1 I A2 I A3 I B II A II B III A III B	1 (4.3%) 8 (17.4%) 2 (4.3%) 10 (21.7%) 8 (17.4%) 11 (23.9%) 3 (6.5%) 1 (2.2%)	0 (0%) 0 (0%) 1 (17.1%) 6 (31.6%) 2 (10.5%) 6 (31.6%) 1 (5.3%) 1 (5.3%)	1 (2.4%) 7 (17.1%) 8 (19.5%) 4 (9.8%) 2 (4.8%) 10 (24.4%) 7 (17%) 0 (0%)	0.113

**Table 4:** Hospital stay and surgery duration.

Variables	Pre-Pandemic	Pandemic	Post-Pandemic	*p*
Duration of surgery (min., M ± SD)	263.4 ± 71.33	242.73 ± 52.74	285.25 ± 71.66	0.244
Pre-operative hospitalization (days, M ± SD)	2.65 ± 1.9	1.37 ± 0.49	2.83 ± 1.7	0.006
Post-operative hospitalization (days, M ± SD)	9.59 ± 4.41	9.16 ± 2.71	11.54 ± 5.74	0.092
Total hospitalization (days, M ± SD)	12.24 ± 4.72	10.53 ± 2.87	14.37 ± 5.83	0.015

## Data Availability

The datasets used and/or analyzed during the current study are available from the corresponding author upon reasonable request.
